# An Unanticipated Worsening of Glycemic Control Following a Mild COVID-19 Infection

**DOI:** 10.7759/cureus.26295

**Published:** 2022-06-24

**Authors:** Ellen M Hardin, Darian R Keller, Taylor P Kennedy, Chad H Martins

**Affiliations:** 1 Internal Medicine, Mercer University School of Medicine, Savannah, USA; 2 Internal Medicine, Memorial Health University Medical Center, Savannah, USA

**Keywords:** covid-19, sars-cov-2 infection, hypertriglyceridemia, hyperglycemia, hemoglobin a1c, outpatient, insulin resistance, glucose metabolism, diabetes, type ii diabetes mellitus

## Abstract

We describe a case of acute-onset worsening of a patient’s previously well-controlled type 2 diabetes mellitus (T2DM) following his recovery from a novel severe acute respiratory syndrome coronavirus 2 (SARS-CoV-2) infection. A 78-year-old male with a three-year medical history of well-controlled T2DM (controlled by diet and metformin) presented to the outpatient clinic to discuss his regularly scheduled six-month lab work. He mentioned having a mild coronavirus disease 2019 (COVID-19) infection lasting one week which required no medical treatment approximately two months before his current visit. His labs, taken one week prior to his current visit, were notable for fasting hyperglycemia, 301mg/dL, and an elevated hemoglobin A_1C_ (HbA_1C_), 11%. A fasting blood glucose level was recorded at his current in-office visit and was found to be 403mg/dL. These findings were not anticipated - our patient reported no change in his meals, medications, or exercise routines. The only notable change he reported between visits was his COVID-19 infection. This case report explores the link between this virus and our patient's exacerbation of his previously well-controlled T2DM. Whether it be through insulin resistance or deficiency (or another unknown mechanism), our patient's prior novel COVID-19 infection could potentially be associated with his unprecedented altered glucose metabolism.

## Introduction

Throughout the coronavirus disease 2019 (COVID-19) pandemic, individuals with diabetes have faced unique challenges. It has been shown that some acute viral respiratory infections have been associated with transient insulin resistance, and growing evidence suggests that COVID-19 may share this association as well [1]. It has also been suggested that infection with this virus may lead to alterations of metabolic parameters that are sustained in the recovery phase. This finding exhibits the possibility of a sustained impact on the patient’s ability to metabolize glucose appropriately [2]. While it is generally understood that infection has the ability to destabilize glycemic control in the acute setting, this potential long-term impact is important for physicians and patients to consider in the outpatient setting. An example of this situation is shown with our patient- a man with a three-year history of well-controlled type 2 diabetes mellitus (T2DM) who presented to the clinic with an unprecedented rise in his hemoglobin A_1C_ (HbA_1C_) to 11%. While he insisted that he had no change to his lifestyle, diet, exercise, or medication, he did report a COVID-19 infection approximately two months prior.

## Case presentation

A 78-year-old male with a three-year medical history of relatively well-controlled, non-insulin-dependent T2DM presented to the clinic to discuss his most recent lab work. Since the onset of his diabetes three years ago, he has been attending regularly scheduled six-month follow-up appointments to discuss labs and management of his conditions. Over the course of his diabetes, he has maintained compliance with his medications, taking metformin as prescribed without needing dosage adjustments. His HbA_1C_ remained in the range of 6.5-7.2%.

Our patient arrived at his clinic visit with no complaints, but he informed the staff that he had been sick with a mild COVID-19 infection since his last visit. He stated that the onset of the illness (confirmed by polymerase chain reaction testing) was approximately eight weeks prior and that the course of the disease lasted about one week. He mentioned no major complications, and he denied requiring steroids or any hospitalization during the course of his infection. He had completed his COVID-19 vaccination (Moderna) series approximately one year before his current visit. Besides this recent illness, the patient mentioned no new changes in his current daily regimen including meals, medications, or exercise. He does not check his blood glucose at home. He denied any new symptoms at his visit, such as polyuria, polydipsia, blurred vision, poor wound healing, fatigue, or lower extremity numbness. 

The patient’s physical examination revealed a well-appearing, well-developed, overweight man in no acute distress. His exam was unchanged from his last visit, except for a mild drop in weight. There was no neurological deficit, sensory deficit, skin changes, or poor vascularization noted. No abnormalities were noted on his diabetic foot exam. The rest of the physical exam was unremarkable.

Laboratory findings (taken one week before his clinic visit) were significant for fasting hyperglycemia (301mg/dL) and an elevated HbA_1C_ (11%). These results were notably different compared to the laboratory results that had been recorded over the past three years (Table 1). After reviewing these findings, the patient’s fasting glucose was re-measured during his current visit and was recorded to be 403mg/dL.

**Table 1:** Pertinent Labs Over Time This table shows our patient's labs that display his glycemic control over time. His infection with COVID-19 occurred approximately two months prior to the most recent values. Reference ranges are as follows: Normal: Hemoglobin A_1C_ (HbA_1C_) <5.7%, fasting glucose <100mg/dL Prediabetes: HbA_1C_ 5.7%-6.4%, fasting glucose 100-125mg/dL Diabetes: HbA_1C_ ≥6.5%, fasting glucose ≥126mg/dL

	3/7/19	9/19/19	2/6/20	8/4/20	2/4/21	8/2/21	2/4/22
Hemoglobin A_1C_ (%)	6.7	7	6.8	6.6	6.8	7.2	11
Fasting glucose (mg/dL)	118	120	141	142	130	145	301

Due to his marked in-office fasting hyperglycemia and noteworthy lab results, our patient was given ten units of Fiasp® insulin (Novo Nordisk Inc, Plainsboro, NJ) during his visit. He was sent back to the lab for measurement of his C-peptide levels, which were recorded as 2.4 ng/mL. His home medications were adjusted: his metformin dose was increased from once to twice daily, and he was begun on a sliding-scale insulin regimen. He was educated on proper insulin dosing and insulin pen usage and felt comfortable with this plan moving forward. The patient was also asked to record his blood glucose levels over the next three days with his new medication regimen (Table 2). Due to his persistent hyperglycemia, glipizide was added to the patient’s medication regimen. Following this change, the patient reported adherence to his new medication plan and appropriate blood glucose levels over the next week.

**Table 2:** Patient's Self-Reported Blood Glucose Readings (mg/dL) Following Visit This table shows our patient's blood glucose levels over the three days after his clinic visit. He reported usage of his sliding-scale insulin regimen over these days as well. The reference ranges are as follows: Normal: fasting glucose <100mg/dL, random glucose N/A* Prediabetes: HbA1C 5.7%-6.5%, fasting glucose 100-125mg/dL, random glucose N/A* Diabetes: HbA1C >6.5%, fasting glucose >126mg/dL, random glucose > 200mg/dL *There are currently no random blood glucose values indicating normal glucose tolerance or prediabetes, however, >200mg/dL indicates diabetes.

	Morning (Fasting)	Before Lunch	Before Dinner	Before Bedtime
Day 1	283	388	191	315
Day 2	210	125	134	328
Day 3	218	214	177	219

## Discussion

Throughout the COVID-19 pandemic, many studies have begun exploring the link between this viral infection and diabetes. It has been suggested that the relationship between the two conditions is bidirectional: the presence of hyperglycemia predisposes a COVID-19 patient to worse outcomes (i.e. increased potential for ICU admission, need for ventilation, the chance of mortality), and the presence of COVID-19 infection leading to issues with glycemic control (new-onset hyperglycemia/diabetes, worsening glycemic control in pre-existing diabetes) [3]. 

New-onset hyperglycemia, worsening of pre-existing hyperglycemia, or even ketoacidosis has been seen in COVID-19 patients in an acute, inpatient setting [4]. The issue of hyperglycemia also has been seen to persist in some patient populations following both serious and mild cases of COVID-19 infection, indicating a possible long-term impact of the virus [5]. One prospective study observed patients while in the hospital and then followed up three and six months after discharge. They found that reduced insulin sensitivity persisted and affected patients’ glucose metabolisms six months after the onset of COVID-19 instead of improving over time during the recovery period [1]. Another study that followed up with COVID-19 patients two months after discharge found an alteration in patients’ hormone profiles demonstrating persistent insulin resistance, similar to the hormone profile of individuals with T2DM [2]. A third study showed multiple cases of patients who had relative control of their T2DM prior to infection but required intensifying insulin regimens several weeks to months after contracting COVID-19 to combat their prolonged hyperglycemia [6].

While it has been established that COVID-19 interferes with one’s ability to metabolize glucose, there is some deliberation about the mechanism of hyperglycemia that has been found in infected and recovering patients. The most common theories suggest that it could be driven by insulin deficiency, insulin resistance, or a combination of the two. One study suggests that COVID-19 causes direct viral injury to angiotensin-converting enzyme 2-expressing islet cells, leading to the pancreas’s inability to produce and release insulin when stimulated [7]. A previously mentioned study (that demonstrated a possible similarity in insulin resistance in patients recovering from COVID-19 and those with T2DM) noted that the group recovering from COVID-19 had persistently increased C-peptide levels despite their increasing fasting blood glucose levels, pointing to possible systemic insulin resistance. These researchers also suggested a middle ground between the two aforementioned theories discussing evidence of beta cell hyperstimulation during COVID-19 infection leading to subsequent development of decreased peripheral insulin sensitivity. This hyperstimulation could result in beta cell exhaustion and ultimate demise, leading to a multi-factorial case pinning both insulin deficiency and insensitivity as potential causes of the hyperglycemia seen in COVID-19 patients [2].

It could be assumed that our patient developed some issue with glucose metabolism, shown by the sudden worsening of his relatively well-controlled T2DM. While he was able to control his glucose levels with metformin and a low-carbohydrate diet for three years, an abrupt exacerbation of his T2DM at this level merits further investigation. With nothing changing in our patient’s lifestyle outside of a mild case of COVID-19, one could propose that the loss of his ability to metabolize glucose appropriately was, in some way, related to his infection. If this is true, our case favored that both insulin deficiency and resistance could be affecting his glycemic control. Because this patient’s C-peptide levels remained within normal range despite his markedly elevated glucose levels, it could be inferred that his pancreas remains capable of producing insulin but does so in an insulin-resistant environment. While his pancreas should have been producing further insulin to combat his systemic insulin resistance, his normal C-peptide levels show a potential inability for the pancreas to produce the necessary higher levels of insulin appropriately. While much has yet to be fully understood about COVID-19, our patient is just one example of the perpetuated effects that this virus could cause in recovering patients. The most pertinent limitation to fully understanding this case lies within our inability to monitor our patient in a controlled environment after he contracted and recovered from COVID-19. Along with this, measuring fasting insulin levels would have been useful to utilize the Homeostatic Model Assessment (HOMA) to further and more accurately assess insulin resistance and beta-cell functioning. To fully and appropriately explain this case, it would be beneficial to further investigate the metabolic abnormalities in the recovering COVID-19 patient with more extensive lab panels, and in an environment where medication, diet, and exercise are monitored and regulated.

## Conclusions

The association between diabetes and COVID-19 has been a widely discussed topic throughout the pandemic. Whether through insulin resistance or deficiency, it has been observed that infection with COVID-19 has a dramatic effect on glucose metabolism. While the acute presentation of a hyperglycemic patient infected with COVID-19 has been widely studied, there is still much to be understood about the longer-term effects of this virus. We hope that this case can add to the existing literature about the glycemic issues that can be seen in patients who are infected with or recovering from COVID-19.
